# Targeted Pyroptosis Is a Potential Therapeutic Strategy for Cancer

**DOI:** 10.1155/2022/2515525

**Published:** 2022-11-24

**Authors:** Hao Wu, Dianlun Qian, Xiangfeng Bai, Shibo Sun

**Affiliations:** ^1^Department of Pulmonary and Critical Care Medicine, First Affiliated Hospital, Kunming Medical University, Kunming, China; ^2^Clinical Medicine, Three Class, 2020 Grade, Kunming Medical University, Kunming, China; ^3^Department of Cardiothoracic Surgery, First Affiliated Hospital, Kunming Medical University, Kunming, China

## Abstract

As a type of regulated cell death (RCD) mode, pyroptosis plays an important role in several kinds of cancers. Pyroptosis is induced by different stimuli, whose pathways are divided into the canonical pathway and the noncanonical pathway depending on the formation of the inflammasomes. The canonical pathway is triggered by the assembly of inflammasomes, and the activation of caspase-1 and then the cleavage of effector protein gasdermin D (GSDMD) are promoted. While in the noncanonical pathway, the caspase-4/5/11 (caspase 4/5 in humans and caspase 11 in mice) directly cleave GSDMD without the assembly of inflammasomes. Pyroptosis is involved in various cancers, such as lung cancer, gastric cancer, hepatic carcinoma, breast cancer, and colorectal carcinoma. Pyroptosis in gastric cancer, hepatic carcinoma, breast cancer, and colorectal carcinoma is related to the canonical pathway, while both the canonical and noncanonical pathway participate in lung cancer. Moreover, simvastatin, metformin, and curcumin have effect on these cancers and simultaneously promote the pyroptosis of cancer cells. Accordingly, pyroptosis may be an important therapeutic target for cancer.

## 1. Introduction

Cancer seriously threatens human health worldwide. Based on the most recent data compiled by the International Agency for Research on Cancer (IARC), 19.3 million new cancer cases were diagnosed in 2020 [1]. In 2022, 1,918,030 new cancer cases and 609,360 cancer deaths are predicted to occur in the United States [2]. Lung, stomach, liver, breast, and colon cancer are the top five primary causes of cancer-related death [1]. At present, cancer treatment methods are limited and ineffective, which can only be performed by surgical resection, radiotherapy, or chemotherapy [3]. In addition, the high cost of cancer treatment and the large amount of medical investment also cause a great deal of economic burden to the individual and society [4, 5]. Accordingly, it is crucial to search more efficient and cost-effective ways to treat cancer.

In normal mammalian cells, cells undergo death and renewal as a result of cell aging, infection, or damage, which present homeostasis of cells. Currently, several types of cell death are found, including apoptosis, autophagy, necrosis, necrotic apoptosis, and pyroptosis [6]. Apoptosis and autophagy are important targets of anticancer defense and have been widely studied. Apoptosis is the most common programmed cell death, which is a physiological process involving multiple factors, including the immune response, gene regulation, and signal transduction [7]. Abnormal apoptosis leads to a series of pathological effects, such as tumors, while inducing apoptosis in cancer cells may become a viable therapy for treating tumors [8, 9]. Autophagy is a lysosome-based catabolic process that maintains homeostasis, and the defense capabilities of autophagy are degrading endogenous and foreign substances which are held in vesicles [10]. Autophagy suppresses the development of tumors by eliminating damaged proteins and organelles and avoiding genome damage [11]. Most traditional chemotherapy strategies for cancer are inducing apoptosis or autophagy of tumor cells with erlotinib, paclitaxel, gefitinib, crizotinib, or cisplatin [12–15]. However, studies show that cancer cells undergo infinite proliferation, and cancer cells with a epidermal growth factor receptor (EGFR) wild-type is resistant to chemotherapy drugs [16]; thus, there are few cancer cells which execute apoptosis or autophagy. It indicates that under some circumstances, chemotherapy drugs can not give rise to the apoptosis or autophagy of cancer cells, thus resulting in drug resistance of chemotherapy [17]. Consequently, in order to improve the treatment of cancer, it is crucial to induce another type of cell death. Pyroptosis is a new type of inflammatory cell death which is triggered by the assembly of inflammasomes. The activated caspase-1 results in the cleavage of gasdermin D (GSDMD), the secretion of interleukin-1*β* (IL-1*β*) and interleukin-18 (IL-18), and consequent death of cells [18]. Accordingly, pyroptosis is considered as an important target to treat cancer. This article aims to review the morphological characteristics, signaling pathways of pyroptosis, as well as the relationship between pyroptosis and cancer.

## 2. Characteristics of Pyroptosis

In 1992, a programmed cell death of host macrophages caused by *Shigella flexneri* was mistaken for apoptosis at that time, but this programmed cell death is actually pyroptosis [19]. Cookson and Brennan first proposed the use of “pyroptosis” to describe this new mode of programmed cell death, where “Pyro” refers to the release of proinflammatory cytokines in 2001 [20]. The Cell Death Nomenclature Committee (CDNC) classified cell death into 13 types based on morphological characteristics, among which pyroptosis was listed in 2009 [21], and in 2018, the definition of pyroptosis was further clarified as “pyroptosis is a kind of regulated cell death (RCD) which minutely depends upon the formation of cell membrane pores by gasdermins and often is caused by inflammatory caspase activation” [6].

The morphological characteristics of pyroptosis include cell enlargement, small amount of DNA damage, and chromatin concentration, but the nucleus remains intact [22]. In the early stage of pyroptosis, there is a very specific DNA damage which is different from that of apoptosis. Compared with apoptosis, the intensity of DNA damage in pyroptotic cells is lower, and the nucleus is intact [23, 24]. The pores which are consisted of gasdermin oligomerization appear on the cell membrane and the cell expands when the pyroptosis occurs [25]. In addition, the proinflammatory cytokines such as the IL-1*β* and IL-18 are released through those cell membrane pores; consequently, the pyroptosis continues. Accordingly, pyroptosis is different from apoptosis whose morphological characteristics are manifested in cell shrinkage, DNA degradation, nuclear membrane rupture, and cell membrane integrity [6]. It is generally believed that apoptosis is a regular form of cell death, while pyroptosis is induced by intracellular or extracellular stimulation, such as viral, bacterial, toxin, and chemotherapy drugs [26].

## 3. Signal Pathways of Pyroptosis

The main pathways of pyroptosis are divided into the canonical pathway and the noncanonical pathway according to the upstream signal transducing mechanism [27]. In the canonical pathway, the upstream stimuli lead to the NOD-like receptors (NLRs) which are the members of pattern-recognition receptors (PRRs) that assemble into inflammasomes and then trigger the maturation of pro-caspase-1 to cleave the GSDMD, the pro-IL-1*β*, and the pro-IL-18 [28, 29]. While in the noncanonical pathway, upstream stimuli directly trigger the cleavage of GSDMD by caspase-4/5/11 (caspase 4/5 in humans and caspase 11 in mice) rather than the assembly of the inflammasomes [30].

### 3.1. Canonical Pyroptosis Pathway

The pattern-recognition receptor (PRR) is a vital part of our natural immune system [31]. PRRs recognize pathogen-associated molecular patterns (PAMPs) and damage-associated molecular patterns (DAMPs), degrading pathogens and endogenous substances by assembling into inflammasomes [32–34]. In pyroptosis, it is the NLRs which are the PRRs that assemble into inflammasomes [35]. Except for NLRP1, NLRs contain three usual domains: C-terminalleucine-rich repeat (LRR) domain, central nucleotide-binding and oligomerization (NACHT) domain, and N-terminal pyrin domain (PYD) or caspase activation and recruitment domain (CARD) [23, 36]. The LRR domain has the function of ligand recognition as well as automatic inhibition, the NACHT domain activates signal complexes with the help of ATP, and the PYD domain or CARD domain mediates isotypic protein-protein reciprocities [28, 37]. When immune stimulation occurs, the PYD domain binds NLR to apoptosis-associatedspeck-like protein (ASC) which also incorporates a PYD domain through PYD-PYD interaction [38]. The binding reaction triggers the formation of ASC focal points, which recruit pro-caspase-1 and assemble into inflammasome through CARD-CARD interaction [38, 39]. Subsequently, the assembly of inflammasome results in the transformation of pro-caspase-1 to catalytically activated P10 and P20 subunits, which boosts the activation and maturation of pro-IL-1*β* and pro-IL-18 [40–42].

Activated caspase-1 also facilitates the cleavage of gasdermins besides the pro-IL-1*β* and pro-IL-18 [43]. The gasdermins are proteins which assemble membrane pores by polymerization, thus causing the outflow of cell contents [44]. Most of gasdermins such as gasdermin A (GSDMA), gasdermin B (GSDMB), gasdermin C (GSDMC), gasdermin D (GSDMD), and gasdermin E (GSDME) except DFNB59 have similar structures and functions of forming pores on the cell membrane [44]. When gasdermin is unactivated, the inhibitory C-terminal domain and the functional N-terminal domain connect together to form complete gasdermin, which cannot assemble membrane pores. However, when gasdermin is activated, the N-terminal domain breaks away from the C-terminal domain so that gasdermin assembles membrane pores and triggers further reactions [45]. Increasing evidence suggest that the GSDMD is more important in membrane pores formation compared with other gasdermins [46, 47]. However, the cellular functions and activation mechanisms of gasdermins remain unclear [48].

**Figure 1:**
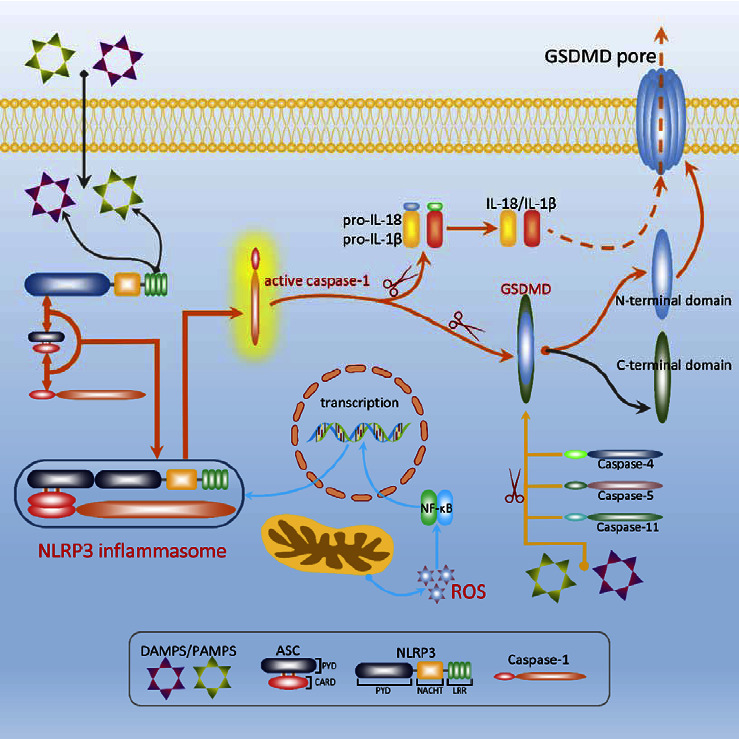
In the canonical pathway of pyroptosis, NLRP3 recognizes stimulus (DAMPS/PAMPS) and binds to the ACS through PYD-PYD interaction and then occurs the formation of ASC focal points which triggers the recruitment of pro-caspase-1 to assemble into NLRP3 inflammasome. In the inflammasome, pro-caspase-1 is cleaved into the active forms, which catalyzes the cleavage of pro-IL-1*β*, pro-IL-18, and GSDMD. Mature GSDMD forms pores on cell membrane, leading to the outflow of cell contents such as IL-1*β* and IL-18, thus exhibiting an inflammatory response. It is noteworthy that the transcription of NLRP3 genes is regulated by the ROS/NF-*κ*B signaling pathway. In the noncanonical pathway of pyroptosis, caspase-4/5/11 directly recognizes stimulus and gets activated, then causing cleaving GSDMD to promote pyroptosis.

The upstream signaling of canonical pyroptosis pathway stimulates the assembly of inflammasome whose PRR is generally NLRP3 [49]. The NLRP3 inflammasome transforms pro-caspase-1 into activatory caspase-1, which promotes the activation of pro-IL-1*β* and pro-IL-18 [28, 37, 50]. At the same time, caspase-1 causes the cleavage of GSDMD and activates the GSDMD [29, 51]. The activated GSDMD forms the membrane pores, which make cell contents, such as IL-1*β* and IL-18, to be released [52, 53]. Consequently, the inflammatory response occurs. Undoubtedly, caspase-1 and GSDMD take up irreplaceable roles in the canonical pyroptosis pathway, and the activation of caspase-1 is mainly sparked by the assembly of NLRP3 inflammasome, so the NLRP3 inflammasome ought to occupy another important role in pyroptosis. It is reported that the reactive oxygen species (ROS)/nuclear factor kappa B (NF-*κ*B) signaling pathway takes part in the activation of NLRP3 inflammasome [54]. ROS promotes the release and activation of proinflammatory transcription factors such as NF-*κ*B, which mainly regulates the NLRP3 inflammasome [55, 56] (Figure 1).

### 3.2. Noncanonical Pathway of Pyroptosis

The downstream of noncanonical pyroptosis pathway is the same as that of canonical pyroptosis pathway and presents GSDMD as the effector protein which causes the formation of cell membrane pores [30, 47]. In addition, the morphological characteristics of the noncanonical pyroptosis pathway are basically the same as that of the canonical pyroptosis pathway. However, the upstream of noncanonical pyroptosis pathway is substantially different from that of the canonical pathway. In the canonical pathway, the assembly of the inflammasomes promotes the maturation of caspase-1, which not only boosts the proteolytic maturation of pro-IL-1*β* and pro-IL-18 but also promotes the cleavage of GSDMD to form cell membrane pores. While in the noncanonical pyroptosis pathway, the caspase-4/5/11 directly receives stimulation, binding to stimulating protein which mainly is the lipopolysaccharides (LPS) of Gram-negative bacteria and then promote the cleavage of GSDMD to form pores rather than assemble into inflammasomes [29, 30, 43, 57, 58]. At the same time, the amino-terminal fragments which are produced in the process of caspase-11 cleaving GSDMD promote the NLRP3 inflammasome and the caspase-1 to be activated, which suggest that the noncanonical pathway crosstalks with the canonical pathway [30] (Figure 1).

## 4. Pyroptosis and Cancer

### 4.1. Pyroptosis and Lung Cancer

Lung cancer (LC) seriously threatens human health worldwide. The survival rate for a period of 5 years is less than 15% [59, 60]. Lung cancer is included into two subtypes which are small-cell lung cancer (SCLC) and nonsmall-cell lung cancer (NSCLC), and the NSCLC accounts for about 85% of lung cancer cases [61]. Chemotherapy is one of the conventional treatment methods of LC [62]. However, chemotherapy is less sensitive and less effective in the therapy of LC [63] because cancer cells have multiple strategies to circumvent or limit apoptosis which is a normal mechanism to protect cells [64]. Accordingly, it is very important for LC to propose new therapeutic strategies.

SCLC accounts for approximately 15% of all lung cancers and is classified as a high-grade neuroendocrine (NE) tumor which has a high death rate and poor prognosis [65]. However, there are only a few studies to explore the relationship between SCLC and pyroptosis. It is reported that chemosensitivity is related to the pyroptosis which is connected with the expression of yes-associated protein (YAP) and GSDME, and the activation of YAP suppresses GSDME expression to enhance the chemoresistance in SCLC cells, while the inactivation of YAP in SCLC tumor cells switches cell death from apoptosis to pyroptosis [66].

In pyroptosis, it is clear that the assembly of NLRP3 inflammasome is closely related to the activation of caspase-1, which is involved in the cleavage and maturation of the GSDMD, and the GSDMD is the executor of pyroptosis. It indicates that NLRP3 inflammasome, caspase-1, and GSDMD are crucial factors in the process of pyroptosis. Accordingly, inducing pyroptosis of NSCLC cells through the NLRP3/caspase-1/GSDMD pathway may be potential targets for inhibiting the tumor progression of NSCLC [54]. Wang et al. demonstrated that caspase-1 was downregulated in NSCLC tumor tissues and found that simvastatin (SIM), an anti-hyperlipidaemia drug, inhibits the growth of NSCLC by activating caspase-1-dependent pyroptosis in xenograft mouse models and in A549 and H1299 lung cancer cells [67]. Additionally, it is suggested that SIM also induces apoptosis by downregulating the cyclin-dependent kinases (CDKs) and matrix metalloproteinases-9 (MMP-9) levels or by inhibiting the activity of proteasome and upregulating p21 and p53 [68, 69]. Further research confirms that SIM induces ROS generation and accumulation in mitochondria and cytosol, thus leading to apoptosis of NSCLC cells [70–72]. The polyphyllin VI (PPVI), a chief saponin extracted from trillium tschonoskii maxim (TTM), induces caspase-1-dependent pyroptosis through the ROS/NF-*κ*B/NLRP3/GSDMD signal axis and inhibits the progression of NSCLC [54]. In addition, PPVI promotes the accumulation of ROS and the cleavage of caspase-3, downregulates the B-celllymphoma-2 (Bcl-2) expression, upregulates the Bcl-2-associated *X* (Bax) and p53 expression, and arrests the cell cycle in *G*2/*M*; thus, apoptosis of NSCLC cells is triggered [73, 74]. Meanwhile, PPVI also exerts the anti-NSCLC effect by inducing apoptosis through the phosphatidylinositol-3-kinase (PI3K)/Akt/mammalian target of rapamycin (mTOR) pathway [75]. The cucurbitacin B (CuB), a compound extracted from muskmelon pedicel, inhibits NSCLC by bounding to toll-like receptor 4 (TLR4) to activate the NLRP3 inflammasome and triggering GSDMD dependent pyroptosis [76]. Also, CuB enhances the mitochondrial ROS to trigger pyroptosis of NSCLC cell [76]. Moreover, CuB induces the apoptosis of NSCLC cells by inhibiting the long noncoding RNA *X*inactive-specific transcript (lncRNA-XIST)/miR-let-7c/IL-6/signal transducer and activator of transcription 3 (STAT3) axis and suppressing the mitogen-activated protein kinases (MAPK) and PI3K pathways [77, 78]. Additionally, CuB arrests the cell cycle of NSCLC cells at the *G*2/*M* phase and downregulates the level of Bcl-2, thus inducing apoptosis via the STAT3 pathway [79, 80]. In addition, CuB induces apoptosis by interfering with EGFR activation and its downstream signal path which includes Akt and extracellular-signal-regulated kinases (ERK) [81, 82]. It is presented that dasatinib (DAS), a multikinase inhibitor, promotes the cleavage and secretion of the GSDMD and GSDME which induce the pyroptosis of A549 cells, which thus inhibits the progress of NSCLC [83]. DAS also induces the apoptosis of lung cancer cells by upregulating the ROS level or downregulating the Bcl-2 family member Bcl-xL [84, 85]. In addition, CD8 (+) T cells require GSDMD for an immune response to NSCLC, while GSDMD deficiency results in the cytolytic capacity of CD8 (+) T cells [86].

The ROS/NF-*κ*B pathway is involved in the expression of NLRP3 inflammasome which indicates that both the ROS and the NF-*κ*B act vital roles in pyroptosis [56, 87, 88]. Likewise, both ROS and NF-*κ*B are targeted to induce pyroptosis in NSCLC therapy [54]. Chalcone, a natural structure, induces pyroptosis by the upregulation of ROS and inhibits the progress of A549 and H1975 cells [89]. Meanwhile, chalcone upregulates the caspase-3, caspase-8, Bax, and ROS and inhibits the cell cycle at the G2/M phase ultimately resulting in apoptosis of A549 cell [90, 91]. Metformin (MET), a biguanide drug, induces pyroptosis of tumors by the adenosine monophosphate (AMP)-activated protein kinase (AMPK)/Sirtuin-1 (SIRT1)/NF-*κ*B/caspase-3/GSDME pathway [92]. The mechanisms are that MET upregulates the AMPK/SIRT1 pathway and increases the expression of NF-*κ*B, activating the cleavage of GSDME by caspase-3 [92]. Moreover, MET induces caspase-3-dependent apoptosis through regulating SIRT1 and activating the c-junN-terminal kinase (JNK)/p38 MAPK pathway [93, 94]. Piperlongumine (PL) analogue L50377, a natural product with less toxicity, is applied to induce pyroptosis of NSCLC through upregulating the level of ROS and activating the expression of NF-*κ*B [95]. It is shown that PL also induces apoptosis and autophagy of NSCLC cells through activating the PI3K/Akt/mTOR pathway, upregulating the microRNA-34b-3p, and downregulating the transforming growth factor beta type I receptor (TGFBR1) [96, 97]. It is reported that 13 d (a modified EF24 with low toxicity) or L61H10 (a thiopyran derivative) may mediate the apoptosis-pyroptosis switch in NSCLC through the NF-*κ*B signaling pathway [98, 99]. Furthermore, EF24 analogues promote ROS generation and accumulation, resulting in apoptosis of NSCLC cells [100, 101].

Apurinic endonuclease 1 (APE1) acts as a key factor in base excision repair (BER) and exerts the function of apurinic sites excision [102]. It is reported that the poor prognosis of NSCLC links with high level of APE1 [103–105]. The NO.0449-0145 (a small molecule compound) improves the condition of NSCLC by inhibiting the expression of APE1 and inducing pyroptosis [106]. Furthermore, Zhu et al. suggested that inhibiting the activation of APE1 leads to elevation of the p53 protein level and increase of the NSCLC apoptosis [107, 108].

Maternal embryonic leucine zipper kinase (MELK) is a carcinogenic kinase and is essential in NSCLC mitotic progression, metastasis by regulating the process of cell death [109]. It is reported that MEIK has overexpression in cancer cells [110]. Tang et al. demonstrated that OTSSP167, as a potent inhibitor for MELK, blocks the *G*2/*M* phase cycle of lung adenocarcinoma (LUAD) cells by inhibiting MELK to trigger the pyroptosis [111]. The inhibition of MELK also decreases its downstream forkhead box protein M1 (FOXM1) activation and Akt expression in lung cancer cells, leading to apoptosis of NSCLC cells [112].

Other member of the gasdermins such as GSDME serves as special targets to induce pyroptosis in NSCLC therapy [113]. It is reported that paclitaxel and cisplatin inhibit A549 lung cancer cells by inducing pyroptosis through the caspase-3/GSDME pathway [114]. DAS induces the pyroptosis of A549 cells by upregulating the level of GSDME [83].

Intracellular LPS induces the pyroptosis through the noncanonical pathway [115, 116]. In addition, LPS may directly lead to regression of some tumor; however, the underlying mechanism remains unclear [117]. Currently, it is suggested that the secretoglobin (SCGB) 3A2, as a multifunctional secreted protein, eliminates human lung adenocarcinoma cells through noncanonical inflammasome pathway mediated by LPS [118]. Human NSCLC cells with SCGB3A2-sensitivity express caspase-4 which is a crucial molecule of the noncanonical inflammasome pathway [118].

It is proposed that the pyroptosis is induced by inhibiting the lncRNA-XIST through the Mir-335/SOD2/ROS signaling pathway, and then the NSCLC is inhibited [119]. In addition, the knockout of lncRNA-XIST gene induces the pyroptosis of tumor cells, which suppresses the growth of NSCLC cells and promotes the chemotherapy sensitivity of cisplatin [120]. Moreover, the upregulation of the p53 inhibits tumor growth by promoting pyroptosis in NSCLC [121]. In addition, 4-hydroxybenzoic acid (4-HBA) leads to the activation of pyroptosis by accelerating the transcription of caspase-1, IL-1*β*, and IL-18 genes in A549 cells [122] (Table 1).

However, the high expression of some key molecules of pyroptosis may not lead to amelioration but results in poor prognosis and deterioration of NSCLC in some specific cases. It is suggested that GSDMC is overexpressed in LUAD patients who have poor prognosis [123]. In addition, the high level of GSDMD do not induce pyroptosis but is associated with aggressive characteristics, such as more advanced tumor-lymph node metastasis (TNM) phase, larger tumor size, and poorer prognosis in NSCLC [124]. Zou et al. suggested that NLRP3 promotes the cell proliferation and the migration of NSCLC [125]. Accordingly, more researches are needed to confirm the role of pyroptosis in NSCLC.

### 4.2. Pyroptosis and Gastric Cancer

Gastric cancer (GC) is one of the most common cancers which is seriously harmful to human health [2, 126]. Accordingly, it is very crucial to search new effective methods to treat GC. Here, we discuss some strategies for the treatment of GC by inducing pyroptosis and apoptosis. The pyroptosis-related risk signals and the pyroptosis-related genes (PRGs) in GC may potentially predict the treatment benefit, the prognosis, the survival of individuals, and their response to immunotherapy [127–131]. Moreover, the pyroptosis-related protein GSDMD may inhibit the cell proliferation of GC, and when GSDME is knocked down, the growth of GC cells is affected [132, 133]. It is demonstrated that the release of ROS by sonodynamic therapy (SDT) treatment induces the pyroptosis of GC cells and plays the antitumor function [134]. It is reported that treating GC cells with famotidine triggers the activation of NLPR3 inflammasomes and leads to the mature and secretion of GSDME and IL-18, resulting in the pyroptosis of GC cells [135]. It is presented that SIM activates caspase-3/GSDME expression and thereby induces pyroptosis of GC [136]. In addition, SIM treatment suppresses the expression of *β*-catenin, inhibits the activation of YAP and NF-*κ*B, and thus promotes the apoptosis in GC cells [137, 138]. Icariin (ICA), an active component from TCM epimedium grandiflorum, inhibits the progression of GC cells by activating the NLRP3 inflammasomes and inducing pyroptosis [139]. Meanwhile, ICA could effectively induce apoptosis via hsa_circ_0003159/eIF4A3/bcl-2 axis to reduce the GC cell activity [140]. It is confirmed that diosbulbin-B (DB) is effective to activate NLRP3-mediated pyroptosis in GC by downregulating programmed death ligand-1(PD-L1) [141]. Furthermore, DB inhibits the proliferation of GC cells by knocking-down cerebellar degeneration-related protein 1 (CDR1) (a type of circular RNA) to promote apoptosis [142] (Table 1).

However, the cytotoxin-related gene A (CagA) protein, an important pathogenic factor of *Helicobacter pylori* (*H. pylori*) [143], promotes the invasion and migration of GC cells by activating NLRP3 inflammasome, while the suppression of *H. pylori*-triggered inflammatory response and the depression of pyroptosis via the ROS/NLRP3/caspase-1/IL-1*β* pathway may suppress the progression of GC [144]. These results indicate that there is a close and complex relationship between GC and pyroptosis, while more researches are necessary in future.

### 4.3. Pyroptosis and Hepatic Carcinoma

Hepatic carcinoma (HCC) is a common kind of cancers which seriously hazards human health [2]. However, increasing researches demonstrate that HCC cells present multiple strategies to achieve drug resistance [145, 146]. Accordingly, it is necessary to search effective strategy to treat HCC. It is confirmed that PRGs such as pyroptosis-related lncRNA may serve as a promising biomarker for HCC patients to predict the prognosis and guide precision drug treatment and immunotherapy [147–151]. Meanwhile, pyroptosis-related proteins especially the GSDMD and the GSDME have the potential to become crucial biomarkers for the diagnosis and prognosis of HCC, which provide a new insight for the development of therapeutic targets [152, 153]. NIMA-related kinase 7 (NEK7) is a serine/threonine kinase which progresses the eukaryotic cell cycle [154]. Knocking-down of NEK7 in HCC cells significantly upregulates the expression of NLRP3, caspase-1, and GSDMD to induce pyroptosis and inhibit the migration of HCC cells [155]. It is revealed that euxanthone promotes pyroptosis in a caspase-dependent manner in HCC cells, and alpinumisoflavone inhibits the growth of HCC cells by inducing NLRP3 inflammasome-mediated pyroptosis [156, 157]. Miltirone, a derivative of phenanthrene-quinone isolated from the root of *Salvia miltiorrhiza* Bunge, promotes the accumulation of intracellular ROS and induces the GSDME-dependent pyroptosis of HCC [158]. Likewise, crizotinib (CRIZO) increases ROS in HL-7702 cells to promote pyroptosis and inhibit HCC [159]. Furthermore, CRIZO induces apoptosis and suppresses the proliferation of HCC cells by inhibiting the phosphorylation of the anaplastic lymphoma kinase (ALK), Akt, and ERK [160]. In addition, Cannabidiol (CBD), a cannabis sativa constituent, may induce pyroptosis via caspase-3/GSDME pathway to inhibit the growth of HCC cells in vivo and in vitro [161]. Moreover, CBD arrests the *G*0/*G*1 phase in the cell cycle and induces mitochondrial-dependent apoptosis in HCC cell lines [162]. It is confirmed that MET induces the pyroptosis by promoting forkhead box protein O3 (FOXO3) expression and activating NLRP3 transcription to suppress the progression of HCC cells [163]. Meanwhile, MET induces apoptosis in HCC through the AMPK/p53/p38/miR-23a/FOXA1 pathway or PI3K/Akt/mTOR pathway [164–166]. Furthermore, MET induces the downregulation of Bcl-2 in HCC cells to enhance apoptosis [167]. It is demonstrated that curcumin (CUR) induces pyroptosis in HspG2 cells by increasing ROS [168]. In addition, CUR may inhibit the growth of HepG2 cells by promoting the P53-dependent apoptosis [169]. Moreover, CUR triggers mitochondrial apoptosis in HCC cells by inhibiting the PI3K/Akt/glycogen synthase kinase-3*β* (GSK-3*β*) signaling pathway [170, 171]. 17*β*-estradiol (E2) is a kind of hormonally active compounds [172]. It is suggested that E2-induced activation of the NLRP3 inflammasome may serve as a suppressor in HCC progression [173]. In addition, E2 may promote apoptosis in HepG2 cells by increasing FOXO3 phosphorylation and inducing oxidative stress [174]. Furthermore, E2 inhibits the proliferation of HCC cells through downregulation of IL-6/STAT3 signaling and arresting cell cycle at the *G*2/M phase [175]. It is confirmed that berberine, a kind of isoquinoline alkaloids, inhibits the progression of HepG2 cells by inducing caspase-1-dependent pyroptosis both in vitro and in vivo or promoting apoptosis through the NF-*κ*B/p65 pathway [176, 177]. Additionally, berberine effectively inhibits the growth of HHC cells by inducing adenosine AMPK-mediatedcaspase-dependent apoptosis [178]. These researches highlight the possibilities of inducing pyroptosis or apoptosis for treating HCC and indicate that more studies are needed to clarify the mechanism of pyroptosis in HCC (Table 1).

### 4.4. Pyroptosis and Breast Cancer

Breast cancer (BC) does great harm to women health which ranks second among cancer-related death in women [2]. It is critical to seek a valid treatment strategy for BC. It is confirmed that PRGs may serve as an important prognostic predictor and a chemotherapy target for the treatment of BC [179–183]. In addition, polydatin (PD) downregulates the janus kinase (JAK) 2 and STAT3 levels thus induces pyroptosis, which play an anticancer role in triple-negative BC (TNBC) [184]. Moreover, PD induces apoptosis by suppressing the ROS/PI3K/Akt pathway to inhibit cell proliferation, migration, and invasion of BC cells [185]. It is discovered that cisplatin (DDP) activates the NLRP3/caspase-1/GSDMD pathway to induce pyroptosis of BC cells to exert antitumor effects [186]. Furthermore, DDP induces apoptosis which is connected with downregulating the PI3K/Akt/mTOR signaling pathway in BC cells [187]. It is reported that pyroptosis of BC cells is induced with the AIM2/caspase-3/GSDME axis being activated when BC cells are administrated by dihydroartemisinin (DHA) [188]. Meanwhile, administration of DHA dramatically upregulates the expression of caspase-8/9 and downregulates the level of Bcl-2 and thus results in apoptosis and *G*0/*G*1 cell cycle arrest of BC cells [189]. It is discovered that Nobiletin induces the pyroptosis of BC cells by regulating the MicroRNA-200b/zinc finger gene 1 (JAZF1)/NF-*κ*B pathway [190]. In addition, Nobiletin decreases the expression of Bcl-2, Bcl-xL, increases the expression of Bax, p53, and caspase-3, and inhibits the Akt/mTOR pathway to induce apoptosis of BC cells and suppress the progression of BC [191, 192]. Tetraarsenic hexoxide induces the pyroptotic cell death through the ROS/caspase-3/GSDME axis to suppress the progression of TNBC cells [193]. Likewise, Triclabendazole induces GSDME-dependent pyroptosis by activating caspase-3 in BC cells [194]. Accordingly, pyroptosis provides a new therapeutic approach for patients with BC (Table 1).

### 4.5. Pyroptosis and Colorectal Carcinoma

Colorectal carcinoma (CRC) is the third most common form of cancer in adults which has a poor prognosis and significantly damages the patient's daily life and mental health [2]. Effective therapeutic strategies are urgently needed to achieve better prognosis and therapeutic outcomes of CRC. It is demonstrated that arsenic trioxide (ATO) and ascorbic acid (AA) corporately upregulates the expression of caspase-1 and promotes the formation of inflammasomes to induce pyroptosis in CRC [195]. Meanwhile, ATO inhibits CRC cells growth by inhibiting the activation of telomerase and inducing caspase-3-dependent apoptosis [196]. It is presented that the expression of inflammasomes is increased both in vitro and in vivo after treating CRC cells with decitabine (DAC), which suggests that DAC suppresses the growth of colon cancer by inducing pyroptosis [197]. In addition, DAC increases the expression of miR-133b and triggers the apoptosis in CRC cells [198]. Thus, pyroptosis may be a target of ATO and DAC on CRC (Table 1).

## 5. Conclusion

As a type of RCD mode, pyroptosis plays an important role in several kinds of cancers, whose pathways are divided into the canonical and noncanonical pathway depending on whether formation of the inflammasomes. The canonical pathway is triggered by the assembly of inflammasomes and mainly regulated by the activation of caspase-1. Activated caspase-1 not only promotes the cleavage of effector protein GSDMD but also promotes the proteolytic maturation of proinflammatory cytokines IL-1*β* and IL-18, resulting in the morphological characteristics of pyroptosis. While in the noncanonical pathway, the caspase-4/5/11 directly cleave GSDMD, resulting in the pyroptosis. In addition, pyroptosis is affected by the ROS and NF-*κ*B which influence the upstream pathway of pyroptosis.

Pyroptosis in gastric cancer, hepatic carcinoma, breast cancer, and colorectal carcinoma is related to the canonical pathway, while both the canonical and noncanonical pathway participate in lung cancer. Moreover, simvastatin, metformin, and curcumin have effect on these cancers and simultaneously promote the pyroptosis of cancer cells. Accordingly, pyroptosis may be an important therapeutic target to cancer though the relationship between pyroptosis and a few cancers such as CRC and SCLC remain unclear, and more researches on pyroptosis in these cancers are needed in future.

## Figures and Tables

**Table 1:** The mechanism of different medicines to induce pyroptosis or apoptosis in different cancers.

	Pyroptosis	Apoptosis
*Lung cancer*		
Simvastatin	Activates caspase-1-dependent pyroptosis [67]	Downregulates the CDKs and MMP-9 levels; inhibits the activity of proteasome; and upregulates p21, p53, and ROS [68–72]
Polyphyllin VI	Activates the ROS/NF-*κ*B/NLRP3/caspase-1/GSDMD signal axis [54]	Upregulates ROS, caspase-3, Bax, and p53; downregulates the Bcl-2; and regulates the PI3K/Akt/mTOR pathway [73–75]
Cucurbitacin B	Upregulates the NLRP3 inflammasome, GSDMD, and ROS levels [76]	Inhibits the lncRNA-XIST/miR-let-7c/IL-6/STAT3 axis; suppresses the MAPK and PI3K pathways; and interferes with EGFR activation [77–82]
Dasatinib	Promotes the cleavage and secretion of the GSDMD and GSDME [83]	Upregulates ROS level and downregulates Bcl-2 family member Bcl-xL [84, 85]
Chalcone	Upregulates the level of ROS [89]	Upregulates the capspase-3, caspase-8, Bax, and ROS and inhibits cell cycle at the *G*2/*M* phase [90, 91]
Metformin	Activates AMPK/SIRT1/NF-*κ*B/caspase3/GSDME pathway [92]	Regulates SIRT1 and activates the JNK/p38 MAPK pathway [93, 94]
Piperlongumine	Upregulates the level of ROS and activates the expression of NF-*κ*B [95]	Activates the PI3K/Akt/mTOR pathway; upregulates the microRNA-34b-3p; and downregulates the TGFBR1 [96, 97]
EF24	Mediates the apoptosis-pyroptosis switch through the NF-*κ*B signaling pathway [98]	Promotes ROS generation and accumulation [100, 101]
L61H10	Mediates the apoptosis-pyroptosis switch through the NF-*κ*B signaling pathway [99]	
NO.0449-0145	Inhibits the expression of APE1 [106]	
OTSSP167	Blocks the *G*2/*M* phase cycle by inhibiting MELK [111]	
secretoglobin3A2	Activates the noncanonical inflammasome pathway mediated by LPS [118]	
4-Hydroxybenzoic acid	Accelerates the transcription of caspase-1, IL-1*β*, and IL-18 genes [122]	

*Gastric cancer*		
Simvastatin	Activates caspase-3/GSDME expression [136]	Suppresses the expression of *β*-catenin and inhibits the activation of YAP and NF-*κ*B [137, 138]
Icariin	Activates the NLRP3 inflammasomes [139]	Regulates the hsa_circ_0003159/eIF4A3/bcl-2 axis [140]
Diosbulbin-B	Activates NLRP3-mediated pyroptosis by downregulating PD-L1 [141]	Downregulates the level of CDR1 [142]

*Hepatic carcinoma*		
Crizotinib	Accumulates the ROS in cancer cells [159]	Inhibits the activation of ALK, Akt, and ERK [160]
Cannabidiol	Regulates the caspase-3/GSDME pathway [161]	Arrests the *G*0/*G*1 phase in the cell cycle and induces mitochondrial-dependent apoptosis [162]
Metformin	Promots FOXO3 expression and activates NLRP3 transcription [163]	Regulates AMPK/p53/p38/miR-23a/FOXA1 pathway, regulates PI3K/Akt/mTOR pathway, and downregulates Bcl-2 [164–167]
Curcumin	Increases the generation and accumulation of ROS [168]	Promotes the P53-dependent apoptosis and inhibits the PI3K/Akt/GSK-3*β* signaling pathway [169–171]
17*β*-estradiol	Induces the activation of NLRP3 inflammasome [173]	Increases FOXO3 phosphorylation, induces oxidative stress, and downregulates IL-6/STAT3 signaling [174, 175]
Berberine	Induces caspase-1-dependent pyroptosis [176]	Regulates NF-*κ*B/p65 pathway and induces adenosine AMPK-mediatedcaspase-dependent apoptosis [177, 178]
Euxanthone	Promotes pyroptosis in a caspase-dependent manner [156]	
Alpinumisoflavone	Induces NLRP3 inflammasome-mediated pyroptosis [157]	

*Breast cancer*		
Polydatin	Downregulates the JAK2 and STAT3 levels [184]	Suppresses the ROS/PI3K/Akt pathway [185]
Cisplatin	Activates the NLRP3/caspase-1/GSDMD pathway [186]	Downregulates the PI3K/Akt/mTOR signaling pathway [187]
Dihydroartemisinin	Activates the AIM2/caspase-3/GSDME axis [188]	Upregulates the expression of caspase-8/9 and downregulates the level of Bcl-2 [189]
Nobiletin	Regulates the microRNA-200b/JAZF1/NF-*κ*B [190]	Decreases the Bcl-2 and Bcl-xL; inhibits Akt/mTOR pathway; and increases the Bax, p53, and caspase-3 [191, 192]
Tetraarsenic hexoxide	Activates the ROS/caspase-3/GSDME aixs [193]	
Triclabendazole	Induces GSDME-dependent pyroptosis by activating caspase-3 [194]	

*Colorectal carcinoma*		
Arsenic trioxide	Upregulates the expression of caspase-1 and promotes the formation of inflammasomes [195]	Inhibits the activation of telomerase and induces caspase-3-dependent apoptosis [196]
Decitabine	Upregulates the expression of inflammasomes [197]	Increases the expression of miR-133b [198]

## Data Availability

No data were used to support this study.
